# Effects of Neonatal Dexamethasone Exposure on Adult Neuropsychiatric Traits in Rats

**DOI:** 10.1371/journal.pone.0167220

**Published:** 2016-12-09

**Authors:** Nathanael J. Yates, Donald Robertson, Jennifer Rodger, Mathew T. Martin-Iverson

**Affiliations:** 1 School of Animal Biology, Faculty of Science, The University of Western Australia, Crawley, Western Australia, Australia; 2 School of Anatomy, Physiology, and Human Biology, Faculty of Science, The University of Western Australia, Crawley, Western Australia, Australia; 3 School of Medicine and Pharmacology, Faculty of Medicine, Dentistry and Health Sciences, The University of Western Australia, Crawley, Western Australia, Australia; Chiba Daigaku, JAPAN

## Abstract

The effects of early life stress *in utero* or in neonates has long-term consequences on hypothalamic-pituitary-adrenal (HPA) stress axis function and neurodevelopment. These effects extend into adulthood and may underpin a variety of mental illnesses and be related to various developmental and cognitive changes. We examined the potential role of neonatal HPA axis activation on adult psychopathology and dopamine sensitivity in the mature rat using neonatal exposure to the synthetic glucocorticoid receptor agonist and stress hormone, dexamethasone. We utilized a comprehensive battery of assessments for behaviour, brain function and gene expression to determine if elevated early life HPA activation is associated with adult-onset neuropsychiatric traits. Dexamethasone exposure increased startle reactivity under all conditions tested, but decreased sensitivity of sensorimotor gating to dopaminergic disruption–contrasting with what is observed in several neuropsychiatric diseases. Under certain conditions there also appeared to be mild long-term changes in stress and anxiety-related behaviours with neonatal dexamethasone exposure. Electrophysiology revealed that there were no consistent neuropsychiatric abnormalities in auditory processing or resting state brain function with dexamethasone exposure. However, neonatal dexamethasone altered auditory cortex glucocorticoid activation, and auditory cortex synchronization. Our results indicate that neonatal HPA axis activation by dexamethasone alters several aspects of adult brain function and behaviour and may induce long-term changes in emotional stress-reactivity. However, neonatal dexamethasone exposure is not specifically related to any particular neuropsychiatric disease.

## Introduction

The HPA axis prepares the body to respond to acute anticipatory and reactive stressors including pain, injury, social challenges, and unfamiliar contexts. Activation of distinct neuroanatomical pathways in the HPA axis by stress ultimately results in the release of a variety of hormones including glucocorticoids from the adrenal glands into the bloodstream (predominantly cortisol in humans and corticosterone in rodents). These hormones cross the blood brain barrier and bind to mineralocorticoid receptors (MR) and glucocorticoid receptors (GR) in many brain regions. Although there are protective mechanisms to prevent excessive HPA axis activation in early life, such as placental barriers [1] and mother-infant interactions [2]; sustained stressors and pharmacological treatments may overcome protection mechanisms [3] resulting in changes in stress reactivity and behaviour as adults [4, 5]. The effects of excessive HPA axis activation from early life stress *in utero* or as neonates has consequences extending into adulthood, which are hypothesised to underpin a variety of mental illnesses [3, 4] and neurocognitive deficits [6]. In addition activation of components of the HPA axis, such as perinatal GR activation with dexamethasone, have commonly been associated with adverse neurodevelopmental outcomes [7, 8]. Therefore the use of glucocorticoids in early life, such as commonly done in preterm infants, and excessive glucocorticoid levels during maternal stress have likely impacts on adult development.

Many neuropsychiatric illnesses are characterised by specific changes in brain function that may be indicative the early life stress or HPA axis over activation. Many patients with neuropsychiatric illnesses such as schizophrenia and depression demonstrate HPA axis dysfunction as adults [9, 10] and show changes in GR expression in the brain [11]. Furthermore neuropsychiatric illnesses such as schizophrenia, depression, and anxiety, have risk factors implicating adverse early life environments [12, 13] including maternal infection [14, 15], maternal psychological stress [16], various obstetric complications [17] and maternal famine [18, 19]. Therefore it appears plausible that early life activation of the HPA axis through GR agonism may lead to changes in developmental programming, resulting in changes in brain development, behaviour, and neuropsychiatric phenotypes.

Excessive early life HPA activation leads to key changes in brain structure and behaviours. For example, separation and repeated maternal stress results in changes in adrenal gland size reflecting chronic changes in HPA axis function in offspring [20, 21]. This translates into model specific changes in offspring for maternal separation, alteration in the important neuropsychiatric phenotype of sensorimotor gating [20]; and for maternal stress, poorer spatial memory [21]. However these approaches are limited by the inability to dissect specific developmental responses to activation of distinct components of the HPA axis.

The specific effects GR activation in the HPA axis can be probed using the synthetic GR agonist dexamethasone. Maternal exposure to dexamethasone in rats not only produces changes in offspring GR expression, but also impairs aspects of cognition including memory [22, 23]. However maternal treatment with dexamethasone confounds the effects on the mother and the pup–vehicle-treated pups show behavioural deficits when cross-fostered dams treated with dexamethasone during pregnancy [22]. The confounding effects of maternal dexamethasone exposure may be overcome by directly exposing pups to dexamethasone. Neonatal dexamethasone exposure in rat pups produces long-term changes in HPA-axis responses, brain structure, and motor activity [24, 25]. Regardless of the route of HPA axis activation, it appears that perinatal HPA axis activation leads to long-term changes in stress sensitivity, brain structure, and behaviour. However, it has not been established if the developmental programming effects GR activation produce specific neuropsychiatric phenotypes.

We address the potential contribution of early life GR activation on the development neuropsychiatric phenotypes using neonatal dexamethasone exposure in rats. The presence of neuropsychiatric phenotypes was assessed a battery of well-established tests in patients and animal models of psychiatric disease including: sensory processing and gating [26, 27], amphetamine sensitivity and dopamine receptor expression [28–30], brain activity (electroencephalography (EEG) [31]), and auditory evoked potentials [32–35], novelty seeking and memory [36, 37], and genes implicated in neuropsychiatric illness, brain plasticity, and glucocorticoid function [28, 38, 39]. We found several changes in brain function and behaviour, however these did not appear specific to a particular neuropsychiatric phenotype, indicating that GR activation in early life may not be sufficient by itself to elicit a psychiatric phenotype.

## Materials and Methods

### Animals

This study received approval by the University of Western Australia Animal Ethics Committee (Approval ID: RA/03100/1179), and conducted according to Australian and international guidelines. All animals were housed in standard housing, with *ad libitum* supply of standard rat chow and water, with a 12:12 Light:Dark Cycle. Six pregnant Sprague-Dawley dams arrived in animal housing approximately mid-gestation from local supplier and housed within a few hours of departure from the Animal Resource Centre (Murdoch, Western Australia). Parturition day was considered as postnatal day 0 (PND 0). Because of well-established gender and oestrous cycle effects in behavioural studies only male pups were used in this study. Female pups were culled prior to PND 3 using cervical transection. On PND 5 the remaining male pups were assigned to the vehicle control group (CON, N = 21) or to the dexamethasone treatment group (DEX, N = 21) keeping equal numbers for each group within each litter when possible. The dexamethasone solution was 1.5 mg/ml dexamethasone 21-phosphate disodium salt (Sigma-Aldrich, concentration expressed as that of base) dissolved in 0.9% saline. The groups were weighed and injected with either vehicle (0.9% saline) or dexamethasone solution (1.5 mg/kg i.p.) days PND5 to PND10, inclusive. The blood brain barrier is incomplete at this age [40], making it possible for dexamethasone to act in the brain. This age encompasses the rat HPA axis hyporesponsive period of PND 4–14 when glucocorticoid levels are normally kept very low through mother-pup interactions [41]. Dexamethasone concentration is based upon previous studies showing long-term mild changes behaviour, brain size and HPA axis activity [25, 42–44] and on dose-responses that produce alteration in PPI [45]. Direct pup exposure was chosen due to the potentially confounding effects of dexamethasone on maternal care in maternal exposure models [22]. Injections were performed between 14:00 and 16:00 each day. A handling-only control group was not included: previous studies using similar injection regimes have found no behavioural, body weight, or brain growth differences between vehicle-injected and handled controls in adult rats [24, 46]. The pups were monitored and weighed daily until PND14 and then weekly thereafter. On PND24 the pups were weaned and housed in pairs, with one DEX and one CON treated animal when possible. Regular monitoring revealed all animals were healthy and showed no signs of suffering throughout the study.

### Experimental Design

Starting at postnatal week 13 each animal went through a series of experiments probing the adult phenotype, summarised in Table 1. The same cohort of animals was used throughout the study. The order and design of the experiments was chosen to minimise interference between experiments.

**Table 1:** Ages and timing of experiments. Each animal was tested at each age and for each task listed.

Age	Task
PND5-10	Injections of VEH (N = 21) or DEX (N = 21)
Postnatal Week 13–14	Prepulse inhibition of the startle reflex
Postnatal Week 16	Exploratory behaviour and novel object recognition
Postnatal Weeks 17–18	Electrophysiology, tissue harvesting, qPCR

#### Prepulse Inhibition of the Startle Reflex

Briefly, during weeks 13–14 each rat underwent a series of measurements of prepulse inhibition (PPI) of the startle reflex consisting of a habituation day, amphetamine challenge (AMPH) and vehicle challenge (VEH) days. The challenges of AMPH (dexamphetamine, 2 mg/kg dissolved 2 mg/ml in 0.9% saline) or VEH (0.9% saline, 1 ml/kg) were administered 10 min prior to commencement of the experiment.

Each experiment was separated by one day with the amphetamine and vehicle challenge days counterbalanced, and only the amphetamine and vehicle challenge days were included in analysis. The PPI protocol used a variety of stimulus intensities with and without a prepulse to generate stimulus intensity response magnitude curves (SIRM) [47, 48]. The protocol also included trials at the beginning and end of each day testing session to assess habituation of PPI and the startle response (see S1 File for full details).

#### Exploratory behaviour

During postnatal week 16 all animals were assessed for exploratory behaviour and novel object recognition. At least 15 min prior to the exploratory behaviour assessments, rats were habituated to the test room which was moderately illuminated. The apparatus was a 2 m long enclosed Perspex tunnel (approx. 15 cm x 15 cm square) divided into a light (transparent perspex) half and a dark (black perspex) half. Each rat was placed at the farthest end of the dark half of the tunnel shut at both ends with sliding Perspex doors. Over the course of 10 min the number of times the rat crossed between the light and dark zones, latency to first cross, and the total amount of time the rat spent in the dark was recorded. A cross was considered to have occurred when all four paws moved into a different zone. The data were divided into two 5 min blocks (first 5 min, last 5 min).

#### Open-field exploration

The day after exploratory behaviour assessment each animal was placed in an open-field chamber (L x W x H, 19.5 x 31.5 x 12 cm) marked with a 6 x 6 grid, for 5 min and recorded with a video camera with the experimenter outside the room.

#### Novel Object Recognition

The days following open-field exploration rats were individually habituated to an open field box for 3 days (l x w x h, 18.5 x 18.5 x18 cm). During the training session, two identical objects were placed in the top right and left corners of the open field and the rat was allowed to explore for 5 min. They were returned to their home cage for 1 h, and then again placed in the open field containing one duplicate of the original objects and one novel object for 5 min. The time spent interacting with the novel object compared with the familiar object during the retention test was used as a measure of recognition memory. The testing box and objects was cleaned between trials using 70% ethanol to mitigate odour cues. The types and locations of the objects were counterbalanced.

### Electrophysiology

Between 17 and 19 weeks postnatal, rats received a comprehensive test battery of electrophysiological assessments using EEG and auditory evoked potentials for measures that are often abnormal in neuropsychiatric disease including in schizophrenia. Briefly the animals were anaesthetised with 60 mg/kg pentobarbital sodium with additional doses as needed of 30 mg/kg i.p as monitored by foot-withdrawal reflex, eye-blink reflex and breathing. The rat’s head was secured in a stereotaxic frame (Stoelting Co.) and small silver ball electrodes (0.75 mm diameter) were placed on the brain surface through small burr holes in the frontal sinus (common reference), above the left and right auditory cortex (active electrodes, 4.5 mm posterior, 3.5 mm lateral to bregma) and a common indifferent was placed above the cerebellum (common ground). Electrodes used insulated silver wire. ECG was monitored using needle electrodes placed on each forepaw. Each electrode pair was passed through a DAM-50 differential amplifier and acquired using a 4-channel PowerLab system (AD Instruments), see Table 2 for filter settings related to each evoked potential measure. Testing occurred in a heated sound attenuated room and upon a heating pad. Following electrode placement ear bars were removed the head resting on the bite bar. The stimuli were presented binaurally through a centrally placed open field speaker. Auditory stimuli and digital triggers were generated by custom written software (AEP Generator version 1.0, NJ Yates) using LabView 2011 (National Instruments) and presented by an external USB soundcard sampled at 96 kHz (Creative USB X-Fi). Data were presented and acquired online using LabChart 7 (AD Instruments). Offline analysis was performed using MATLAB 2012a (MathWorks Inc.) and LabChart 7.

**Table 2:** Electrophysiology recording and testing parameters. The auditory stimulus parameters, recording parameters, and outcome measures or each electrophysiology protocol.

Protocol	Stimulus	Acquisition	Measures
ABR	0.1 ms click trains; 100 ms interstimulus interval; 80 dB to 25 dB SPL in 5dB steps	Filter 300–3000 Hz; Sampling 40 kS/S; 500 averages; 2ms prestimulus and 10ms poststimulus windows	Auditory threshold based upon peak IV/V disappearance. Also measures auditory function from cochlear nerve to inferior colliculus.
MAEP/LAEP	0.1 ms clicks; 1.5, 0.5, 0.25s interstimulus interval; 60dB Above threshold	Filter 1–3000 Hz; 50 ms pre-stimulus to 200 ms postimulus window; 100 averages	Habituation effects due different ISIs; activation of auditory structures.
P50	Paired 0.1 ms clicks; Interstimulus interval 500 ms or 250 ms; 10 s intertrial interval; 50dB above threshold	Filter 1–300 Hz; 100 ms prestimuls and 900 ms poststimulus window; 60 averages	Sensory gating of the click pairs, where the first click (Conditioning stimulus, C) is larger than the second click (Test stimulus, T). Gating is dependent of interstimulus interval
ASSR	0.1 ms click trains; 500 ms long at 40 and 20 clicks per second	Filter 1–300 Hz; 400 averages 160ms pre-stimulus and 899 ms postimulus window	Measures of cortical synchrony and power: Phase-locking factor; mean trial power; evoked-trial power
EEG	No stimuli	10 mins recording; 2 mins analysed; filter 0.1–1000 Hz	Measures of waveform complexity, entropy and power
ECG	No stimuli	10 min recording at the end of the experiment	Waveform peak durations and intervals, heart rate, and heart rate variability

The experimental protocols are summarised in Table 2 and full description of the experiment methodology is found in supplementary data (S2 File). Briefly, auditory evoked potentials (AEPs) have previously been described as abnormal in human patients with schizophrenia. The AEPs assessed were auditory brainstem responses (ABR) to establish hearing sensitivity, middle latency auditory evoked potentials (MAEP) to assess auditory habituation, P50 response to assess sensory gating, auditory steady state response (ASSR) to assess brain synchrony and phase locking (additional information and example recordings in S2 File). Ten minutes of ECG and EEG were also recorded to assess measures of heart rate variability and waveform complexity which are abnormal in schizophrenia [31] (additional information in S2 File). We excluded any rat that showed ABR thresholds above 43 dB SPL (CON N = 1, DEX N = 4) due to potential confounds of surgical trauma and limitations in speaker output levels. The electrophysiology recording session was approximately 2 hours per animal.

At the end of the electrophysiology protocol, the burr holes over the auditory cortices were extended laterally, anteriorly and posteriorly to expose the temporal lobes. Core biopsies of the temporal lobes in the auditory cortex region were taken. These were immediately placed in ice-cold RNAse-free 0.3 ml eppendorf tubes and into a -80°C freezer for later analysis. Immediately prior to tissue collection animals were euthanized with intraperitoneally injection of Lethabarb (160mg/kg, concentration 325 mg/ml sodium pentobarbitone, Virbac animal health).

### Gene expression in the auditory cortex

Briefly, total RNA was extracted from the frozen samples of auditory cortex and quantitative real-time PCR was used to assess the expression of 5 genes of interest implicated in mental illness: the dopamine receptor 1 (DRD1) and dopamine receptor 2 (DRD2); the GABA cell marker Gamma Amino Decarboxylase 1 (GAD1); NMDA receptor subunit GRIN2B; and a marker of glucocorticoid activity TSC22. These genes were normalised using the geometric mean of 3 references genes: TBP, HPRT, and GAPDH. The auditory cortex was chosen in order to potentially relate changes in auditory evoked potentials with gene expression, is sensitive to dopaminergic-mediated plasticity [49]. Full details of the sample preparation and protocol is presented in supplementary material (S3 File).

### Analysis

Electrophysiology data were originally stored in LabChart (AD Instruments, version 7.3.7) then exported to MATLAB (Mathworks, version 2012a) for processing. Data and statistical analyses were performed using R studio (version 0.97.551) and R (version 3.0.1), with packages ez (version 4.1–1) and plyr (version 1.8), GraphPad Prism (version 5.04, GraphPad Software Inc.) and Microsoft Excel 2007. Non-parametric tests were Wilcoxon ranked sum. Parametric tests were mixed model 2-way ANOVAs and ANCOVAs using the ezANOVA function in R. Statistical post-hoc tests were Holm-Bonferroni corrected t-tests with significance levels at α = 0.05, unless otherwise stated. Because only the effects or interactions related to dexamethasone treatment were of interest, differences observed due to recording side in electrophysiology experiments are only reported if other main effects or interactions are present, otherwise only results from the left ACx are shown. Brief statistical reporting is provided in the text, however full analysis can be found in supplementary data (S1, S2 and S3 Files).

## Results

### General Observations and Physiology

Between 12 and 20 pups were born per litter (6 to 12 males). Prior to dexamethasone injections there were no pre-existing differences in pup weight, but following injections DEX animals showed persistent lower weights. In addition, the dexamethasone pups showed eye opening at least 1 day earlier than the vehicle control group (days, DEX = 11.67 ± 0.33, CON = 13.5 ± 0.34, t = 5.966, df = 5, p < 0.01). Differences in fur growth were also apparent within the pre-weaning stage, DEX animals showing patchy and course hair growth whilst the fur of VEH animals was fine and smooth.

### Behavioural Phenotype

**Fig 1:**
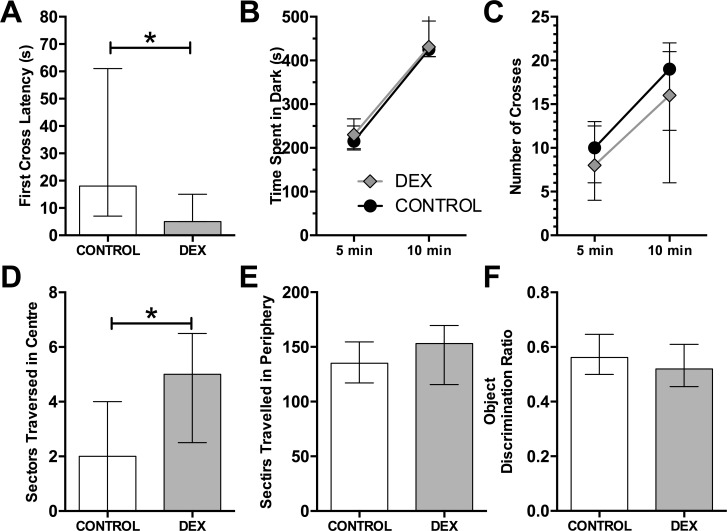
Behavioural testing outcomes with dexamethasone treatment. The light-dark tunnel (A to C), open field (D & E) and novel object recognition (F). Light-dark tunnel: A) Latency to first cross from dark to light side, B) total time spent in dark in 5 and 10 min, C) total number of crosses between light and dark. Selected open field exploration data: D) & E) show the Median number of centre and peripheral sectors travelled respectively. F) Discrimination ratio from novel object recognition. Statistics were Wilcoxon ranked sum. There were no significant differences. Data represent median ± inter-quartile range. Significant differences: * p < 0.05.

When placed in the light-dark tunnel the DEX group showed a significantly shorter latency to first cross to the light zone (Fig 1A, Wilcoxon ranked sum test, W = 139.5, p < 0.05). However all other measures from the light-dark tunnel were not significantly different from the VEH group (Fig 1B, Time in dark; Fig 1C, Crosses). The light zone in this test is usually considered to be anxiogenic, indicating that DEX animals show a low anxiety phenotype in this environment.

When placed in an open field, dexamethasone treatment resulted in no differences between the groups for total sectors travelled, rearings, defecation, or time spent immobile (all p > 0.10). However, the number of sectors traversed in the centre was greater in the DEX group than in the CON group (Fig 1D, p < 0.05) as was the time spent in the Centre (p < 0.01). The number of sectors traversed in the periphery was not different (Fig 1E, p > 0.10). The number of transitions from periphery to centre was not significant (p = 0.055). This phenotype of increased centre activity is often considered to be indicative of a low anxiety animal.

There were no changes in novel object recognition between groups (Fig 1F: discrimination ratio, time looking at novel and habituated objects, and percentage of time interacting with either object, all p > 0.10). Detailed statistics of all behavioural tests are presented in supplementary data (S1 File).

### Sensorimotor response and gating

The acoustic startle response was assessed in each animal with amphetamine or vehicle in a counterbalanced design to determine the effects of neonatal dexamethasone on sensorimotor integration and acoustic sensory processing. All startle response values are expressed as a percentage of maximum observed response in the control group. Within each treatment group and drug condition, there were no significant correlations between body weight and any startle parameter and therefore, the variable body weight was not included as a factor in further analysis.

#### Habituation of Startle Responses and PPI

**Fig 2:**
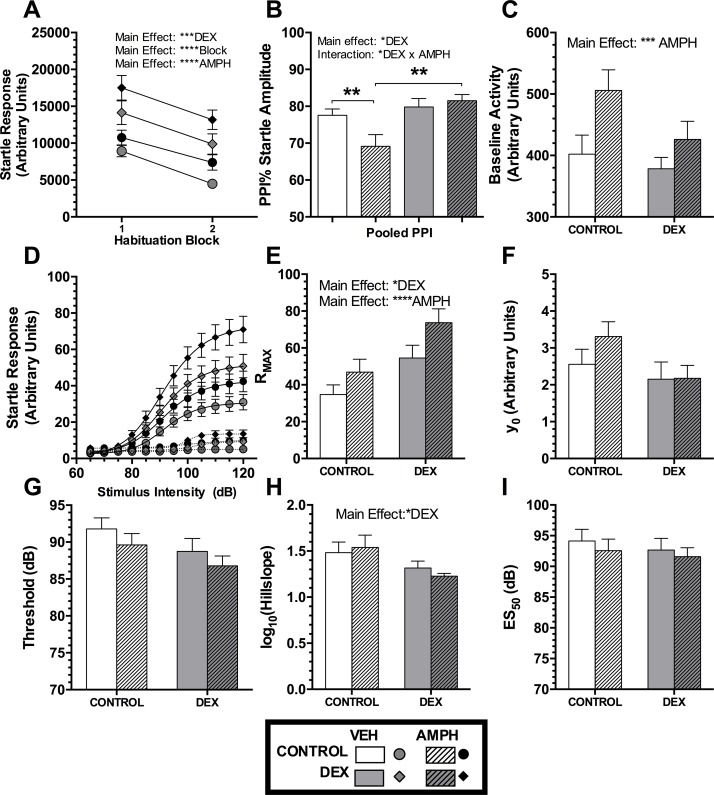
Results from the prepulse inhibition of the startle reflex experiments. The habituation block of trials at the beginning and end of the experiment A) startle response to the habituation trials and B) the PPI response with and without dexamphetamine (AMPH). C) The baseline activity without startle pulses. D) The group average stimulus intensity–response magnitude curves for the startle response trials, with prepulse (dotted lines) or without prepulse (solid lines) curve fits. The parameters from curve fits to the startle only condition are shown in E) to I). E) R_MAX_ is the predicted maximum startle response and F) y_0_ is the theoretical minimum startle response. G) The Threshold of sound intensity required to elicit a startle response. H) the logarithm of Hillslope, which is a measure of maximum velocity of the curve, where acceleration is 0. I) ES_50_ the fitted value where startle response is half of maximum. Data were analysed using mixed model 3 or 2-way ANOVAs, as appropriate. Main effects of Dexamethasone treatment (DEX), Habituation block (Block), Amphetamine treatment (AMPH) and interactions are indicated by text within each panel. Bars show means and SEM. Comparison bars show post-hoc analysis Holm-Bonferroni t-tests. Significance values: *p<0.05, **p<0.01, ***p<0.001, ****p<0.0001.

During the course of an experiment it is normal for ASR and PPI values to change due a process of habituation, which itself provides information on sensorimotor processing mechanisms. Habituation was tested by comparing the ASR and PPI values from the beginning and the end of the experiment in response to maximum startle stimulus intensity (Fig 2A). Analysis using a 2-way mixed design ANOVA showed that in both groups, there was habituation, and that AMPH increased startle response (both p < 0.0001). Dexamethasone treatment increased overall startle (p < 0.001) but did not interact with habituation or AMPH (p > 0.10). Therefore dexamethasone injections did not affect habituation of startle or the effects of amphetamine on startle habituation.

We also examined the habituation of PPI of the startle reflex magnitude. PPI was not affected by habituation (p > 0.10), and there were no interactions between habituation and any other main effects (p > 0.10). A lack of habituation effects on PPI allowed the use of ANOVAs on averaged PPI across habituation blocks. Using pooled PPI there was a significant main effect of dexamethasone (p < 0.05), and an interaction between dexamethasone group and AMPH treatment (p < 0.05). Holm-Bonferroni corrected t-tests of selected comparisons revealed that AMPH injections decreased PPI in the CON group, relative to both the CON+VEH and the DEX+AMPH groups (Fig 2B). This indicates that the dexamethasone group PPI is unchanged by AMPH administration whilst the CON group PPI is reduced by AMPH. Full statistics are in supplementary material (S1 File).

#### Startle Intensity—Response Magnitude Curves

Analysis for the SIRM characterisation phase of the experiment used averaging of the startle responses followed by a curve fitting procedure. All startle responses were expressed as a percent of the maximum observed Control animal response before the fitting procedure. The averages and curve fits, and of the parameters in Fig 2D to 2I. Full statistical reporting found in supplementary material (S1 File).

#### SIRM Responses to Startle Pulse

Changes in SIRM characteristics due to neonatal exposure to dexamethasone are shown in Fig 2. There were no differences in baseline activity between the dexamethasone treatment groups (Fig 2C, p > 0.10), although AMPH caused elevated activity in both groups (p < 0.001). The DEX group demonstrated elevated maximal startle magnitudes (R_MAX_ in Fig 2E, p < 0.01) and so did AMPH treatment (Fig 2E, p < 0.0001). For R_MAX_ AMPH treatment affected startle responses equally in both groups (no interaction, p > 0.10). The dexamethasone group had a reduced log Hillslope (Fig 2H, p < 0.05); which indicates a reduced maximum velocity in the dynamic phase of the SIRM curve and reduced sensorimotor coupling. There were no main effects or interactions for y_0_, Threshold, or ES_50_ (Fig 2F, 2G & 2I, all p > 0.05).

#### SIRM Measures of Sensorimotor Gating

Sensorimotor gating was assessed using the percent inhibition of R_MAX_ by prepulses (for motor gating), or the difference scores between SIRM parameter fits to startle alone and to prepulse trials (including ES50, a measure of sensory gating; and hillslope a measure of sensorimotor gating). There were no differences in any measures of sensorimotor gating (all p > 0.05). Unlike the results in the habituation blocks, there was a non-significant change for R_MAX_ PPI of AMPH selectively disruption of PPI in controls (DEX group and AMPH interaction, p = 0.0947). There was no effect of DEX on shifting of Threshold (p = 0.0506), full statistics reported in supplementary material (S1 File).

In summary the DEX group had elevated startle responses, despite having lower weight. When treated with VEH there was no evidence of disrupted sensorimotor gating in the DEX group compared to controls. However, we found evidence for insensitivity to the disruptive effects of AMPH on PPI.

### Electrophysiology

#### Auditory Brainstem Responses

Auditory brainstem responses were acquired from each rat. After determining thresholds, we assessed the amplitudes and latencies of individual peaks in the ABR waveform offline. There were no differences in peak amplitudes, latencies, ratios of amplitudes, or inter-peak latency for left or right auditory cortex (ACx) recordings at 30 dB above threshold (all p > 0.05, data not shown). Example of ABR traces from a CON and DEX animal are shown in supplementary material (S2 File).

#### Middle and late latency responses

Middle latency auditory evoked potentials showed responses with three main components (example traces in S2 File). There was a consistent positive peak occurring approximately 10 ms post-stimulus (P1), followed by a large negative peak (N1) at approximately 20 ms. There was an additional broad peak occurring approximately between 40–60 ms (P2) and another broad peak ~70–90 ms (P2/2). The negative component between P2 and P2/2 was often ill defined, and thus was not included in analysis. All measurements are peak-peak values, with the exception of P2/2, which is measured from the N1 peak value.

**Fig 3:**
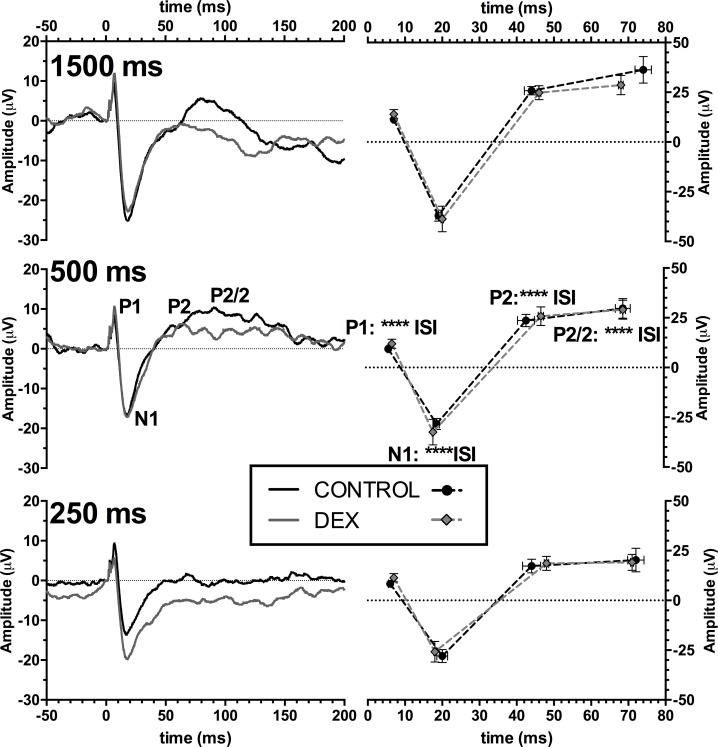
Middle latency auditory evoked potential (MAEP) outcomes. Group grand mean averages for MAEP responses (Left Column) at different inter-stimulus intervals (interval indicated in bold text) assessing the fatigue effects of evoked the peaks. (Right Column) peak latency and peak-to-peak amplitudes at different interstimulus intervals. Analysis used mixed model 2-way ANOVA, main effects of interstimulus interval (ISI) indicated above the relevant peak in the center panel. Significant differences: **** p < 0.0001.

Middle latency responses were used to assess possible differences in habituation to auditory stimuli with different interstimulus intervals (ISIs). We analysed the average traces of each animal in which there were high quality traces at each ISI (250, 500 and 1500 ms). Each peak amplitude was affected by interstimulus interval (main effect for each peak, p < 0.05), indicating that habituation effects were taking place with decreasing ISI. There were no main effects of dexamethasone group in our animals. Summary amplitude and latency data are shown in Fig 3.

#### P50 Auditory Gating

**Fig 4:**
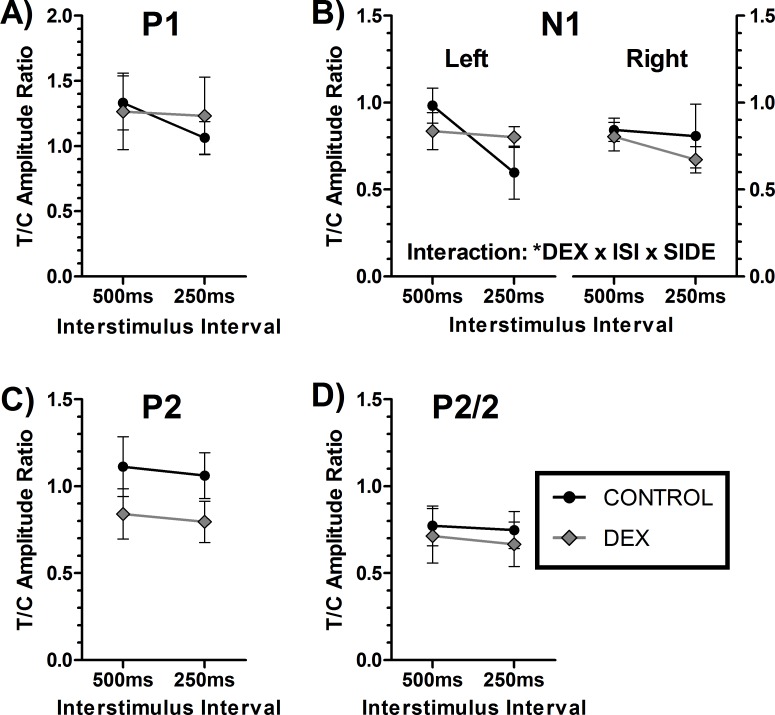
The P50 experiment test-conditioning peak gating ratios. Panels A) to D) show the response from the peaks P1, N1, P2 and P2/2 respectively from the auditory cortex. All panels show left auditory cortex recording except panel B) which shows the left and right ACx, due to interactions with the side of recording. Plots show mean ± SEM. Analysis used 3-way mixed model ANOVA, with possible main effects and interactions of Dexamethasone treatment (DEX), Interstimulus interval (ISI) and recording side (SIDE). Significant differences: * p<0.05.

The use of a paired click paradigm showed a relatively stable waveform. Peaks were identified using the same method as used for MAEPs, and showed no baseline differences in amplitudes (data not shown). An example trace is shown in supplementary material (S2 File) with inset identifying the short latency ABR trace. Measures of sensory gating are measured as a ratio of the test/conditioned response. The group average traces are shown in supplementary material (S2 File). For reasons of brevity, side differences in P50 responses are only discussed when other differences are also present. There were no differences in peak amplitudes for the conditioning response (Data not shown, all p>0.10). There were significant differences in C and T amplitudes for N1, P2, and P2/2, indicating that gating was taking place for these peaks (all p < 0.05), but not for P1 (p > 0.05). For C-T gating ratios (Fig 4), with the exception of N1 for the gating ratios, there were no main effects or interactions with DEX (all p > 0.05). For N1 there was a significant interaction with interstimulus interval, dexamethasone group and recording side (Fig 4B, p < 0.05), though post-hoc hoc tests did not reach significance (full statistics in S2 File).

#### Auditory Steady-State Response

Trains of click stimuli were presented at 20 or 40 Hz, with a train duration of 0.5 s. Three measures were derived from the resulted waveforms. (1) Evoked trial power (ETP) which is the fast Fourier transform (FFT) power of the averaged traces. (2) Mean trial power (MTP) is the averaged FFT for each trace, resulting in a measurement of frequency power that is independent of the phase of the signal. (3) Phase locking factor (PLF), which is a measure of phase alignment of the signals between trials, independent of power. Each of these measures used a moving Hanning tapered FFT window, divided by the average value in the pre-stimulus period, and 20*log_10_ constant applied. This is the most sensitive method for normalising signals within each animal group [50].

**Fig 5:**
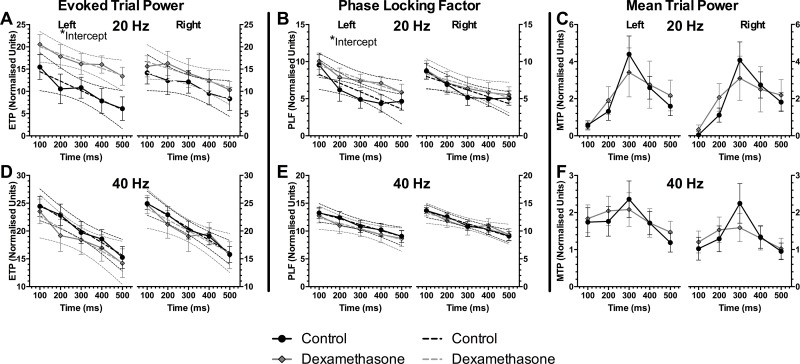
Auditory steady-state response (ASSR) outcomes. Evoked trial powers (ETP, A & D), phase locking factors (PLF, B & E) and mean trial powers (MTP, C & F) to ASSR stimulus trains at 20 Hz (top row) or 40 Hz (bottom row). Left axes for each panel shows recordings from the left auditory cortex, and right axes from the right side. Dotted lines indicate confidence linear regression curve fits, error bars show ± SEM, significant differences between groups: * p<0.05. Analysis for ETP and PLF used regression statistics whilst MTP used 2-way ANOVAs.

The grand means for each of these measurements are represented as heat-maps (S2 File). In order to quantify the differences, averages of the responses are made over several time frames. In this case, averages of the response in 100 ms intervals at the frequency of the ASSR stimulus are shown. Because there was an approximately linear decay in PLF and ETP with time, linear regression was applied to these measures to determine if there was a difference in the rate of habituation or desynchrony (slope of the fit), or overall magnitude of the response (intercept) (Fig 5).

There were no differences in ETP or PLF for 40 Hz ASSR (Fig 5D & 5E). There were increases in the dexamethasone group for PLF and ETP (intercept) for 20 Hz ASSR (p < 0.05), from the left side recording only (Fig 5A & 5B). The slopes were not significantly different for any ETP or PLF condition (p > 0.05).

Unlike PLF and ETP, mean trial power was analysed using 2-way ANOVAs due to the large peak occurring at ~250ms (Fig 5C & 5F) resulting in a highly non-linear shape of the response, preventing the use of linear regression models. The 2-way ANOVA failed to reveal any differences between the groups at 20 Hz or 40 Hz (Fig 5D, 5E, 5F: all p > 0.05). Complete ASSR statistics are reported in S2 File.

#### EEG and ECG signal analysis

**Fig 6:**
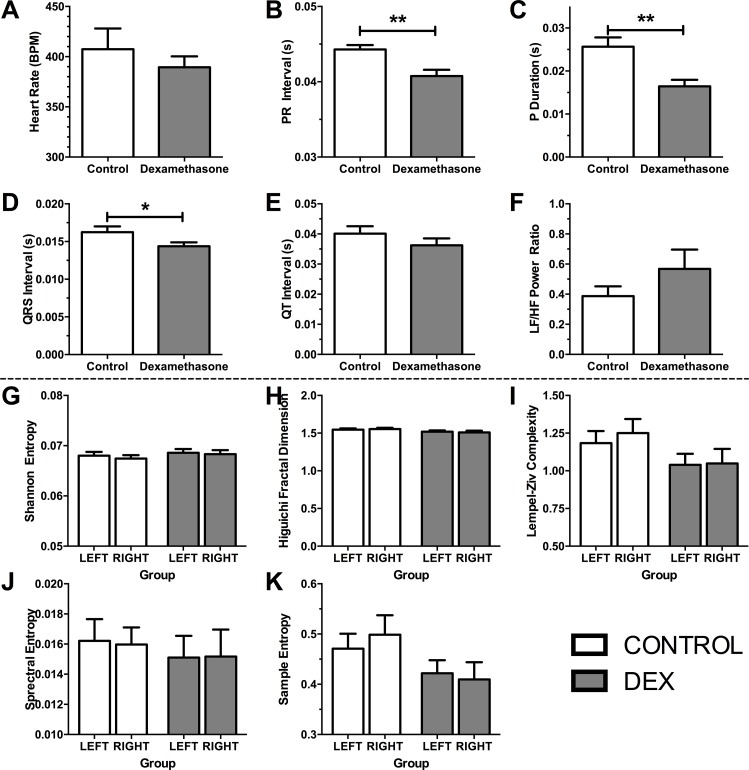
ECG (top panels) and EEG outcome measures (bottom panels). Top panels: Measures of heart function from 10 min ECG trace. A) Heart rate beats per min, B) PR Interval, C) P duration, D) QRS Interval, E) QT Interval, F) Heart rate variability Low Frequency / High Frequency ratio. Definitions: Beats per minute, BPM; Low Frequency / High Frequency Power Ratio, LF/HF Power Ratio. Analysis using 2-tailed t-tests. Bottom panels: Complexity and entropy analysis of EEG data from the left are right auditory cortex. G) Shannon Entropy, H) Higuichi Fractal Dimension, I) Lempel-Ziv Complexity, J) Spectral Entropy, and K) Sample Entropy. Bars show mean + SEM. Significant differences: * p<0.05, **p<0.01.

Adult ECG revealed several changes in cardiac and/or autonomic physiology when DEX and VEH groups were compared. ECG data were obtained for 10 min at the end of the experiment electrophysiology experiment from each animal, and the components of the trace were examined for changes in cardiac function (Fig 6). Briefly, there were no changes in heart rate (Fig 6A, p > 0.10), but indications of faster atrial and ventricular contractions (P-wave duration, Fig 6C, p < 0.01; QRS interval, Fig 6D, p < 0.05), and shorter atrial-ventricular contraction interval (Fig 6B, p < 0.01) with dexamethasone. There were no significant differences in QT interval (Fig 6E, p > 0.10), or an indicator of parasympathetic-sympathetic tone, heart-rate variability (Fig 6F, p > 0.10).

The EEG data between groups were analysed for several measures of entropy and complexity, as defined by Sabeti and colleagues [31] as well as comparing power band spectra (Delta, Theta, Alpha, Beta, Gamma). Using repeated measures 2-way ANOVA we found that there were no differences in any measure of entropy, complexity, or power in EEG spectra. Measures of entropy and complexity are shown in Fig 6G to 6K, and full statistical reporting in supplementary material (S2 File).

### Quantitative Real-Time PCR (qPCR)

**Fig 7:**
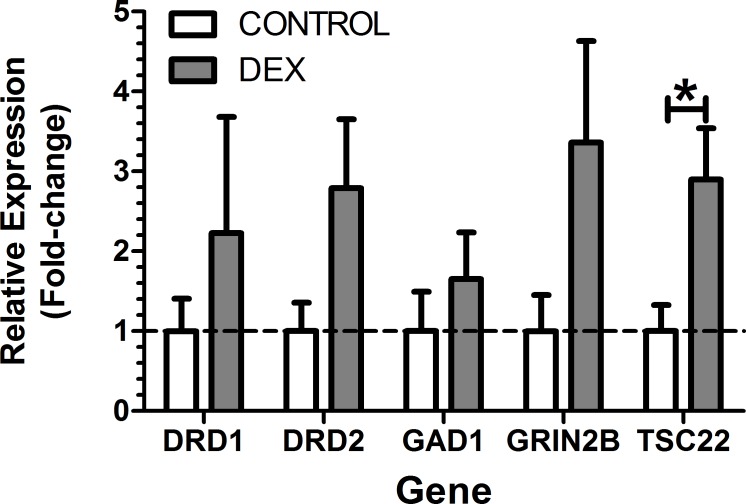
Relative gene expression from the left Auditory Cortex (ACx) compared to control + SEM. Analysis used Student’s t-test except for DRD1 which used Mann-Whitney U. Significant differences: * p < 0.05.

qPCR was performed on the left ACx for gene expression (Fig 7), following the termination of the electrophysiology experiments. The relative expressions of dopamine receptor 1 (DRD1), dopamine receptor 2 (DRD2), glutamate decarboxylase 1 (GAD1, a marker of GABAergic neurons), or GRIN2B (encodes for NMDAR subunit 2B), TSC22 (a marker of glucocorticoid activity) were examined and were selected *a priori* for analysis. Standard student t-tests were performed on all genes except DRD1, which violated homogeneity of variance (F-test, p < 0.05), For DRD1 Mann-Whitney U was used. There was a 2.9 fold increase in expression of TSC22 in DEX (p < 0.05). There were no other significant differences between groups (p > 0.05). Complete qPCR statistics are reported in supplementary material (S3 File).

## Discussion

Our study evaluated the association of neonatal HPA axis activation during development with neuropsychiatric phenotypes in the adult using neonatal exposure to the synthetic glucocorticoid GR agonist, dexamethasone. We found no association between neonatal dexamethasone exposure and neuropsychiatric phenotypes, rather in some cases phenotypic traits were found that were opposite of those observed in mental illness such as decreased amphetamine sensitivity and increased startle reflex responses. Our results suggest that GR activation in early life is not sufficient by itself to elicit a specific and robust psychiatric phenotype, but may produce long-term behavioural outcomes related to attention, stress, and amphetamine/dopamine sensitivity.

### Phenotypes Associated with Neonatal Dexamethasone

Whilst we did not find any specific neuropsychiatric phenotypes with neonatal dexamethasone exposure, it appears that it leads to developmental programming effects on attention and stress. Previous research confirms some of our findings of no link between prenatal dexamethasone exposure and neuropsychiatric phenotypes such as normal or increased sensory gating, and normal latent inhibition [51, 52]. Sensory gating, is the ability to subconsciously contextually filter stimuli, and is modulated by amphetamine sensitivity, usually in the context of dopamine function. Sensory gating is abnormal in a variety of neuropsychiatric disorders, with reported changes in startle reflex response and/or prepulse inhibition (PPI) for: anxiety disorders [53], autism spectrum disorders (ASD) [54], bipolar disorder [55], post-traumatic stress disorder (PTSD) [56, 57], and in schizophrenia [27]. Sensory gating in our rats using PPI revealed that our glucocorticoid exposed group have normal basal sensory gating and exaggerated startle reactivity. Similar neonatal dexamethasone studies have also found exaggerated startle response [25]. These results indicate that neonatal dexamethasone treatment may result in hyper-vigilance or stress response, especially in high stress environments. Our exaggerated startle and normal PPI may be similar to what is found with patients with PTSD [56, 57], in children with parent history of anxiety [58], and in specific contexts adults with anxiety disorders [53]. A relationship between exaggerated startle and hypervigilance is supported by evidence showing anxiolytic drugs may decrease startle reactivity without altering PPI [59].

Whilst baseline PPI was normal with neonatal dexamethasone, our study indicates that neonatal exposure to dexamethasone in our animals resulted in reduced amphetamine sensitivity. Elevated dopamine levels induced by treatments including dexamphetamine reduces PPI in healthy humans [47] and animals [60], yet dexamethasone-treated animals show no reduction in PPI with amphetamine administration. Because the effects of amphetamine on PPI are dependent on dopamine [61, 62], our results suggest that dexamethasone-treated animals have reduced dopamine sensitivity as adults, rather than increased amphetamine and/or dopamine sensitivity observed in schizophrenia, bipolar, and depression [28, 63, 64].

### Neonatal Glucocorticoids and Dopamine

Changes in glucocorticoids activity following neonatal dexamethasone exposure may explain changes in dopamine sensitivity. Elevated glucocorticoid activity in our study is suggested by increased *TSC22* levels [65] and there is a large body of evidence linking glucocorticoids with dopamine. Glucocorticoids decrease locomotor sensitivity to amphetamine injected into the nucleus accumbens [66], pre-treatment with dexamethasone decreases hyperlocomotion response to cocaine and amphetamine [67, 68], and multiple dose regimens of dexamethasone in adult mice decrease locomotor sensitivity to dopaminergic agonists including dexamphetamine [68]. Therefore an increased baseline glucocorticoid activity in dexamethasone treated rats in our study would explain the reduced PPI sensitivity to dexamphetamine.

Changes in dopamine sensitivity may be due to changes in the striatum programmed by neonatal dexamethasone. The striatum is a locus of dopaminergic disruption of PPI but not startle magnitude [69], and its volume is reduced in rats treated with high levels of dexamethasone as pups [42]. Whilst our results are consistent with changes in striatum dopamine, it is important to note that markedly different effects of glucocorticoids on the dopaminergic system are observed depending on dose level, timing and sex of the animals making unequivocal comparisons between studies difficult [70].

More broadly speaking, prenatal stressors have been associated with changes in dopaminergic nuclei structure, innervation and dopamine release [71, 72] as well as a reduction in dopamine D2 receptor binding [73]. Similarly other prenatal stress models show changes in dopamine sensitivity and receptor expression [74].These changes occur in areas commonly associated with mental health, but are not consistent with a specific psychiatric phenotype. Therefore glucocorticoid exposure, and perinatal stress more generally, may not produce neuropsychiatric phenotypes, but may alter development in regions of the brain and in systems associated with mental health such as striatal dopamine.

### Neonatal Dexamethasone and Brain Activity

There were few indications that neonatal dexamethasone produces long-term changes in resting or evoked brain activity. Various prototypical neuropsychiatric traits were absent in our study such as reduced habituation (startle reflex [55, 75, 76], MAEPs [34, 56, 76–79]), other measures of sensory gating, and more nascent areas of research such as signal complexity analysis [31]. Changes in the left auditory cortex auditory-steady-state response (ASSR) were present, which is consistent with reports indicating that prenatal stress affects lateralization of dopamine cerebral function [80]. ASSR lateralisation is diminished in schizophrenia [81] yet our changes do not represent schizophrenia-like traits [82] or any other clear neuropsychiatric phenotype to our knowledge. Changes with cortical activity have been reported with perinatal dexamethasone. Tapered neonatal dexamethasone in rats alters cortical spreading depression velocity (an evoked propagating wave) in mature animals, but not cortical potentiation [83]. Whilst maternal exposure to dexamethasone in fetal sheep produces changes in EEG power bands lasting at least several days *in utero* [84], however it is unknown if these changes extend into adulthood, or for more than a few days. Therefore, perinatal glucocorticoids may have effects on brain function, but they do not appear to enduring changes specific to neuropsychiatric traits in electrophysiology measures of brain function.

### Neonatal dexamethasone produces a phenotype of altered stress reactivity

Although neonatal dexamethasone exposure failed to generate marked neuropsychiatric phenotypes, it produced several other long-term changes in other measures of behaviour which may be interpreted mild changes in stress reactivity. In addition to changes to startle reflex described above, the rats treated with dexamethasone showed reduced neophobia, (Light-Dark box and open field outcomes), but with no indications increased exploration. However, studies using moderate doses of neonatal dexamethasone on PND7 or tapered doses have found reduced open field activity at maturity [25, 85], often considered a sign of increased stress or anxiety. Other studies from mice given tapered dexamethasone show no changes in total locomotion, but contrary to our results shows reduced open field centre time, and reduced in light region of a light-dark box [52]. Whilst different dexamethasone dosages and age at assessment may explain differences with our study, neonatally treated rats show no sign of anxiety in low stress environments regardless of dexamethasone treatment regime [25, 85]. Additionally, the effects of neonatal dexamethasone are somewhat dependent on neonatal handling [86]. As such, the order and timing of our experiments may have affected our outcome as multiple tests may have caused lowered stress by generalised habituation to testing and handling. This may explain why some of our most robust results occur in the startle reflex testing indicating elevated vigilance or stress, which were performed in a high-stress environment at the beginning of our testing protocol when experimental habituation would be low.

Whilst our study did not directly measure HPA activation, our findings are consistent with the hypothesis that neonatal GR activation and stress result in long-term changes of the stress response, and alterations in the response to dopaminergic agents. Persistent upregulation of *TSC22 (GILZ)* in adult rats treated neonatally with dexamethasone suggests elevation of glucocorticoid activity. In addition to its role in immune activation [87], *TSC22* is a sensitive marker of glucocorticoid activity [65]. Previous studies showed that neonatal dexamethasone exposure results in normal basal corticosterone, but blunted responses to novel contexts [24, 88, 89] and elevated response to stress [46]. Studies using neonatal dexamethasone generally show normal cognitive function such as in maze tests [42], with differences only observable under stressful conditions such as active avoidance tasks [90] which may explain the broadly normal responses in our study. The exception to low stress environments in our study was the PPI experiment, which is also where the most obvious differences occurred.

One minor weakness in our study is the lack of a non-injected control group, as the injection procedure in newborn animals can itself be considered a stressor [2]. However, previous studies using low-dose dexamethasone PND3-6 have demonstrated no differences between vehicle and handled controls [24, 85]. In addition our study cannot rule out the possibility of neonatal mineralocorticoid receptor (MR) activation leading to neuropsychiatric phenotypes. Neonatal exposure to corticosterone leading to MR activation can result in context-specific changes in behaviour and learning that are dissociable from GR effects [91]. While dexamethasone is a GR specific agonist, its administration leads to a decrease in corticosterone release, and therefore a reduction in MR activation [92]. The phenotype of neonatal rats treated with MR agonists and antagonists would provide useful insight into the role of developmental stress in neuropsychiatric illness.

## Conclusion

Perinatal dexamethasone exposure is particularly relevant to clinical applications, it is commonly used and trialled in various dosage regimes to promote lung and other organ system development in preterm infants, with varied neurodevelopmental outcomes [8, 93]. We have demonstrated that neonatal glucocorticoids may produce enduring changes in stress reactivity and in dopamine release. However, a “one-hit” neonatal glucocorticoid model of neuropsychiatric illness may be inadequate. It is likely that complex environmental and genetic interactions are important in the development of neuropsychiatric traits.

Whilst prolonged GR activation like that used in our study revealed no specific neuropsychiatric phenotypes, there are indications in the literature that prenatal stress can produce neuropsychiatric phenotypes, but only with unpredictable stressors [12]. In addition genotype-specific effects may be occurring in the general population. For example, dopamine receptor DRD4 genotype interacts with prenatal stress to predict aggressive behaviour and cortisol levels in young adults, whilst prenatal stress alone is not a predictor of behaviour [94].

The growing evidence for the developmental programming effects on neonatal dexamethasone and glucocorticoid exposure highlights the need for future research to consider how genetic and environmental factors interact with early life stress to determine neuropsychiatric risks and phenotypes.

## Supporting Information

S1 File Detailed information and statistics for behavioural testing. This file contains detailed protocols for prepulse inhibition of the startle reflex, and statistics for prepulse inhibition of the startle reflex, open field, and light-dark tunnel tests.
(DOCX)

S2 File Detailed information, example traces, protocols, and statistics for electrophysiology tests. The file contains extra data for all electroencephalography (EEG), electrocardiography (ECG), and auditory evoked potentials (AEP) experiments.
(DOCX)

S3 File Additional real-time quantitative PCR information. Contains full statistical analysis and methods.
(DOCX)
