# Supplementary material for: Effects of Neonatal Dexamethasone Exposure on Adult Neuropsychiatric Traits in Rats
Source: PLoS One. 2016 Dec 9;11(12):e0167220. doi: 10.1371/journal.pone.0167220 (PMC5147874; doi:10.1371/journal.pone.0167220)
Supplement: S1 File — This file contains detailed protocols for prepulse inhibition of the startle reflex, and statistics for prepulse inhibition of the startle reflex, open field, and light-dark tunnel tests. (DOCX) [file pone.0167220.s001.docx]

#

## SUPPLEMENTARY DATA

# S.1 Behavioural Testing

*S1.1 Prepulse Inhibition of the Startle Reflex: Design and Analysis*

We assessed prepulse inhibition of the startle reflex (PPI) during postnatal weeks 13 or 14 using a full stimulus intensity reflex magnitude (SIRM) protocol [1, 2]. Briefly, animals were handled briefly for 7 days before the experiment. In total each rat was tested 3 times for PPI, with a one-day break in between each session. The first day was a habituation day where each animal was tested but not given any injections. In the second and third sessions each rat was given dexamphetamine (AMPH 2 mg/ml i.p., in a volume of 1 ml/kg) or equivalent volume of vehicle (saline) injection (VEH) immediately prior to testing.

Max Startle

Max PPI

**Stimulus Intensity – Response Magnitude Trials**

Fig A. Prepulse inhibition of the startle reflex study design. At both the beginning and end of the experiment there were a series of 5 trials with maximum startle stimulus intensity (Max Startle), designed to assess the habituation of startle response. Next to these trials were a random mix of maximum startle stimulus intensity 5 trials with and without prepulse (Max PPI) designed to assess the habituation of the PPI response. The middle series of trials had 4 blocks of trials with each block containing each possible combination startle intensity and prepulse intensity used for the Stimulus Intensity – Response Magnitude (SIRM) curve fits.

Prior to the experiment all animals were habituated to 65 dB background noise for 10 min. The prepulse intensity was constant at 75 dB SPL, stimulus onset asynchrony of 50 ms, and inter-trial interval 10-20 s. Startle stimuli consisted of stimuli between 60 – 115 dB in 5 dB increments. Each stimulus combination with and without prepulse was repeated 4 times. All stimuli were 50 ms long with 0 ms rise-fall-times. In addition to these trials there were 5 trials at maximum stimulus intensity to assess the effect of, as well as 5 maximum stimulus trials with and without prepulse to assess the habituation of the startle response and of PPI. Besides injections, all animals were treated identically for each day of testing. This is schematically shown in Figure S.1.

Data were analysed offline as described in [2]. Briefly, curve fits were applied to the average startle magnitudes to estimate several parameters describing the stimulus intensity – response magnitude (SIRM) characteristics using Eq. (1) for SIRM and Eq. (2). Where y = startle response magnitude, x = startle pulse intensity in dB. R_MAX_ = startle maximum asymptote predicted by the curve, y0 = y-axis intercept. ES_50_ is the fitted value SPL (dB) where the response is predicted to be half of maximum. Hillslope = maximum velocity where acceleration is 0 (i.e., the rate of change at the ES_50_).

Eq. (1) $y=R_{max}+ \frac{(y_{0}-R_{max})}{1+{(\frac{x}{{ES}_{50}})}^{Hillslope}}$;

Eq. (2) ${Threshold=ES}_{50}- \frac{R_{max}- y_{0}}{Hillslope}$

The calculation of percent prepulse inhibition of R_max_ was performed using Eq. (3).

Eq. (3) $\%PPI=\frac{{Rmax}_{Startle}- {Rmax}_{prepulse}}{{Rmax}_{Startle}} \times100$

### S1.2 Behavioural Statistical Analysis

| Parameter | Effect | df | F value | p-value |
| --- | --- | --- | --- | --- |
| Startle Response | DEX  Block  AMPH  DEX x Block  DEX x AMPH  AMPH x Block  Block x DEX x AMPH | 1,40  1,40  1,40  1,40  1,40  1,40  1,40 | 15.396  83.792  20.650  0.200  0.608  0.262  0.366 | <0.001  <0.00000001  <0.0001  0.657  0.440  0.612  0.549 |
| PPI% Startle Amplitude | DEX  Block  AMPH  DEX x Block  DEX x AMPH  AMPH x Block  Block x DEX x AMPH | 1,40  1,40  1,40  1,40  1,40  1,40  1,40 | 4.978  1.685  3.200  0.220  7.199  0.233  0.680 | <0.05  0.201  0.081  0.642  <0.05  0.632  0.414 |
| Post-hoc Comparisons of PPI% averaged over Block | | | | |
| Comparison 1 | Comparison 2 | df | t- value | Uncorrected p-value |
| Dexamethasone ^A^ AMPH | Control  AMPH | 40 | 2.885 | **<0.01 |
| Dexamethasone ^A^ VEH | Control  VEH | 40 | 0.710 | 0.482 |
| Control ^B^  AMPH | Control  VEH | 20 | 2.942 | **<0.01 |
| Dexamethasone ^B^ VEH | Dexamethasone  AMPH | 20 | 0.693 | 0.499 |

Table A. Effects of developmental dexamethasone treatment, acute amphetamine challenges, and habituation startle response magnitude and prepulse inhibition. Abbreviations: DEX, dexamethasone group; AMPH, amphetamine condition; VEH, vehicle challenge; Block, habituation block. Statistics are repeated-measures 3-way ANOVAs, or Holm-Bonferroni corrected t-tests. Significant differences underlined. T-test key: corrected p-values, ** p <0.01; unpaired t-test: ^A^; paired t-test: ^B^.

| Parameter | Startle stimulus alone | | | |
| --- | --- | --- | --- | --- |
|  | Effect | df | F value | p-value |
| R_max_ | DEX  AMPH  Interaction | 1, 40  1, 40  1, 40 | 6.724  23.562  1.19 | <0.05  <0.0001  0.281 |
| y0 | DEX  AMPH  Interaction | 1, 40  1, 40  1, 40 | 3.243  1.006  0.883 | 0.079  0.322  0.353 |
| ES_50_ | DEX  AMPH  Interaction | 1, 40  1, 40  1, 40 | 0.356  0.773  0.027 | 0.554  0.385  0.870 |
| Threshold | DEX  AMPH  Interaction | 1, 40  1, 40  1, 40 | 2.739  2.666  0.006 | 0.106  0.110  0.939 |
| log_10_(Hillslope) | DEX  AMPH  Interaction | 1, 40  1, 40  1, 40 | 6.929  0.020  0.507 | <0.05  0.887  0.481 |
| Parameter | Prepulse Inhibition | | | |
|  | Effect | df | F value | p-value |
| %PPI R_max_ | DEX  AMPH  Interaction | 1, 40  1, 40  1, 40 | 0.604  0.858  2.930 | 0.442  0.360  0.095 |
| ΔES_50_ | DEX  AMPH  Interaction | 1, 40  1, 40  1, 40 | 2.614  0.189  0.472 | 0.114  0.666  0.496 |
| ΔThreshold | DEX  AMPH  Interaction | 1, 40  1, 40  1, 40 | 4.062  0.069  0.379 | 0.051  0.793  0.541 |
| Δlog_10_(Hillslope) | DEX  AMPH  Interaction | 1, 40  1, 40  1, 40 | 0.034  1.356  0.020 | 0.854  0.251  0.890 |

Table B. Effects of developmental dexamethasone treatment and acute amphetamine challenges on startle intensity – response magnitude parameters. Abbreviations: DEX, dexamethasone group; AMPH, amphetamine condition. Statistics are repeated-measures 2-way ANOVAs, with significant differences underlined.

### S.2.2 Open Field and Light-Dark Tunnel

| Parameter | Sum of Ranks  (Control, Dex.) | Mann-Whitney U | p-value |
| --- | --- | --- | --- |
| Centre Distance Travelled | 351.5, 551.5 | 120.5 | <0.05 |
| Periphery Distance Travelled | 407, 496 | 176.0 | 0.268 |
| Total Distance Travelled | 395.5, 507.5 | 164.5 | 0.163 |
| Centre/Periphery Transitions | 377, 526 | 146.0 | 0.055 |
| Number of Rearing | 452, 451 | 220.0 | 1.000 |
| Time Immobile | 453.5, 449.5 | 218.5 | 0.970 |
| Centre Time | 337.5, 565.5 | 106.5 | <0.01 |
| Fecal Boli | 446.5, 456.5 | 215.5 | 0.901 |
| Urine Presence | 462, 441 | 210.0 | 0.697 |
| Light-Dark Tunnel | 619.5, 370.5 | 139.5 | <0.05 |
| Latency to First Cross |  |  |  |

Table C. Comparison of open field behaviour and light-dark tunnel latency in Control and Dexamethasone animals. Statistics are Mann-Whitney U, using Gaussian Approximation. Significant differences are underlined.

| Parameter | Effect | df | F value | p-value |
| --- | --- | --- | --- | --- |
| Time in Dark (s) | Time  DEX  Interaction | 1,42  1,42  1,42 | 0.124  0.444  0.006 | 0.727  0.509  0.938 |
| Number of Light-Dark Crosses | Time  DEX  Interaction | 1,42  1,42  1,42 | 6.654  1.300  0.119 | <0.05  0.261  0.732 |

Table D. Comparisons of 10 minutes of light-dark tunnel behavior using repeated-measures 2-way ANOVAs. Abbreviations: DEX, dexamethasone group; Time, time block (1^st^ 5 min vs. 2^nd^ 5 min). Significant differences underlined.

# Supplementary Data References

1. Scholes KE, Martin-Iverson MT. Disturbed prepulse inhibition in patients with schizophrenia is consequential to dysfunction of selective attention. Psychophysiology. 2010;47(2):223-35. doi: 10.1111/j.1469-8986.2009.00927.x.

2. Yates NJ, Martin-Iverson MT, Rodger J. The role of ephrin-A2 and ephrin-A5 in sensorimotor control and gating. Behavioural Brain Research. 2014;275(0):225-33. doi: <http://dx.doi.org/10.1016/j.bbr.2014.08.061>.
