# Supplementary material for: Effects of Neonatal Dexamethasone Exposure on Adult Neuropsychiatric Traits in Rats
Source: PLoS One. 2016 Dec 9;11(12):e0167220. doi: 10.1371/journal.pone.0167220 (PMC5147874; doi:10.1371/journal.pone.0167220)
Supplement: S2 File — The file contains extra data for all electroencephalography (EEG), electrocardiography (ECG), and auditory evoked potentials (AEP) experiments. (DOCX) [file pone.0167220.s002.docx]

#

## SUPPLEMENTARY DATA

# S.2 Electrophysiology Acquisition and Stimuli

The electrophysiology protocol used an AEP procedure that allows for assessment of function of multiple auditory sensory nuclei from the cochlear to the auditory cortex. The protocol also allowed for determination of sensory gating and brain synchronisation, core deficits in schizophrenia.

### S.2.1 Auditory brainstem response (ABR)

Auditory brainstem responses were determined using 0.1 ms click stimulus trains presented at 10/s, in ten descending 5 dB steps, to determine auditory threshold. The recordings were band-pass filtered from 300 to 3000 Hz with a sampling rate of 40 kHz. Threshold was determined as the SPL where a peak IV/V could no longer be seen within 5dB accuracy. This threshold was then used to scale the sound intensity for other AEP experiments. Animals were excluded from further analysis if threshold was greater than 43 dB SPL due to limitations in sound card output. Testing was based upon Yates et al. [1]. Example traces are shown in Figure S.2.





Fig A. Example Auditory brainstem response (ABR) traces. CONTROL (Left panel) and DEX (Right panel) from 500 click presentations at 80dB SPL.

### S.2.2 Middle-latency auditory evoked potentials (MAEP) and Long-latency auditory evoked potentials (LAEP)

The MAEP occurs anatomically and chronologically shortly after the ABR response. They were evoked by 0.1 ms clicks at 1.5, 0.5 and 0.25 s inter-stimulus intervals (ISI), for a total of 100 trials at each ISI, 60 dB above threshold. The recording covered the range of 50 ms pre-stimulus to 200 ms post-stimulus. Multiple ISI times were chosen to assess the documented habituation/fatigue effects of the MAEP and LAEP potentials in rats [2].

### S.2.3 P50 Response

The P50 potential in humans is a measure of sensory gating in which paired clicks are presented. The first click is called the conditioning stimulus (C), and the second click the test (T) stimulus. The difference between the T and C evoked waveforms is a measure of sensory gating as the response to T often decreases in size compared to C. The stimuli were presented as 0.1 ms long paired clicks separated by 500 ms (C500, T500) or 250 ms (C250, T250) with an inter-trial interval of 10s. The band-pass filter of 1-300Hz was used. A total of 60 sets of stimuli at 50 dB above threshold were averaged. This testing is broadly based upon de Bruin et al. [3]. An example trace showing the individual peaks and quality of the waveform is shown in Figure S.3 and group averages in Figure S.4.

When calculating ratios, if extreme outliers were present (> 2.5 x SD from the mean) the ratio was replaced by ratio calculated from the other recording side. If both side ratios were extreme outliers then neither were included in analysis.





Fig B. Example P50 response trace for the 500 ms ISI interval. Left panel shows the response with peak locations indicated. The right panel shows that shows an expanded view of the ABR waveform from the left panel. The conditioning response is shown with black lines, the test response gray lines.





Figure C. Group averages of the P50 response (averaged over the left and right ACx)**.** Left panels show the response to C-T 500 ms interval, right panels C-T 250 ms interval.

### S.2.4 Auditory Steady-State (ASSR)

ASSR is a method which assesses the brains ability to synchronise to stimulus presentation. The protocol was based upon methods described by Vohs and colleagues [4]. Monophasic 0.1 ms click square waveforms of were presented at 40/s or 20/s for 500 ms long trains with a 1 s ISI and 400 trials for each recording. Single trial EEG data were recorded with a stimulus epoch length of 899 ms, with 160 ms pre-stimulus recording for baseline. The data were then transformed into the frequency and phase domain by discrete fast Fourier transforms (DFFT) using a Hanning-tapered 128 ms sliding window, with 10 ms steps. The frequencies analysed were 10-80 Hz, with a 1 Hz resolution. All spectrograms were corrected by dividing the values by the pre-stimulus baseline, applying a 20*log_10_ transform. Mean trial power (MTP), evoked trial power (ETP) and phase-locking factor (PLF) were assessed for each condition [5] for both left and right sides. The data were quantified by averaging the response from 0-100, 100-200, 200-300, 300-400 and 400-500 ms at the frequency of stimulation. Group-averaged heat maps of this data are shown in Figure S.5.


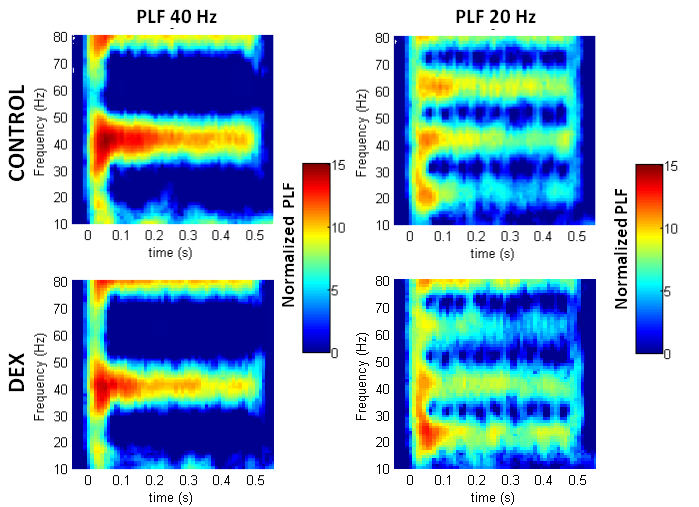

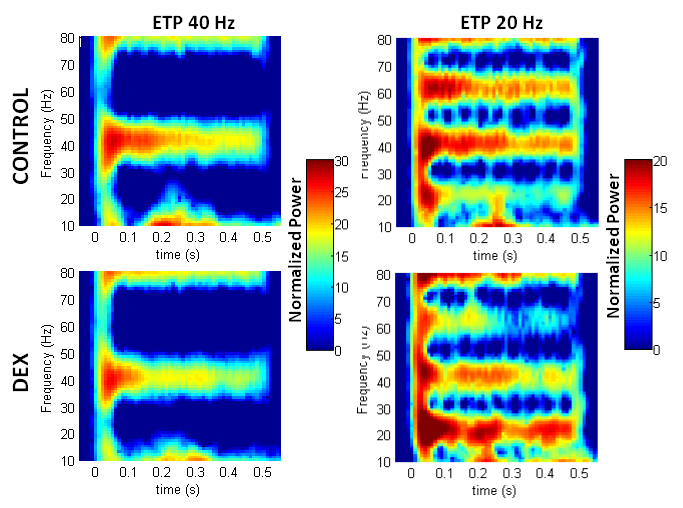

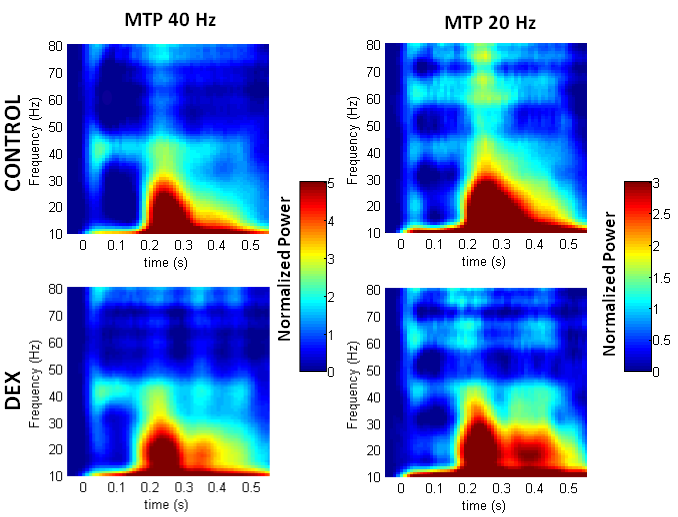


Fig D. Group averages of auditory steady state responses. Auditory click trains presented at 40 and 20 Hz, recorded from the left auditory cortex. A) Evoked trial power (ETP), B) phase locking factor (PLF), and C) the mean trial power (MTP).

### S.2.5 EEG and ECG

Following completion of AEP protocols, 10 min of raw EEG and ECG data were obtained, sampled at 10000 samples/s, with 0.1-1000Hz band pass filtering, and down-sampled to 500 samples/s offline, and filtered with a 4^th^ order Butterworth band pass filter 1-50Hz. EEG data were analysed using methods based upon Sabeti and colleagues [6]. Briefly, each trace was visually inspected for a 2 min interval without artefacts, and normalised by dividing the signal by the standard deviation. Due to the non-stationary nature of EEG signals the trace was divided into 5 s blocks and several measures of entropy was determined including: Shannon Entropy, Spectral Entropy, Approximate Entropy (code by Kijoon Lee, © 2012, from Mathworks online), Lempel-Ziv complexity (code by Quang Thai, © 2012, from Mathworks online), Higuichi Fractal Dimension (code by Salai Selvam, © 2011, from Mathworks online). The average of the 5 s blocks was used in further analysis. Briefly, the parameters used for analysis were: Shannon entropy, 50 bins, upper and min amplitudes 2.5 SD of signal; spectral entropy, 1-50 Hz range with 1 Hz frequency bins; Lempel-Ziv complexity, 0’s and 1’s classified based upon above or below mean; Higuichi Fractal-Dimension, maximum dimension length for comparision was 50; and for sample entropy dimension was 4, tolerance of similarity was 0.2 SD.

The use of ECG in the protocol allowed monitoring of anaesthesia depth. Ten min of ECG from each animal was analysed offline for waveform intervals, heart rate, and heart rate variability (HRV) (LabChart 7.3.7, Modules: ECG Analysis 2.3.2, HRV 1.4.2). HRV frequencies were based upon methodology of Kuwahara and colleagues ([Kuwahara et al., 1994](file:///C:\Users\20242116\AppData\Local\Local%20Settings\AppData\Local\Temp\l%20%22_ENREF_7%22%20\o%20%22Kuwahara,%201994)) using R-R intervals as the measures of heart rate.

### S.2.6 P50 Waveform Statistics

| Peak Ratio | Effect | df | F value | p-value |
| --- | --- | --- | --- | --- |
| P1 | Group  ISI  Side  Group x ISI  Group x Side  Condition x Side  Group x ISI x Side | 1, 35  1, 35  1, 35  1, 35  1, 35  1, 35  1, 35 | 1.498  0.004  0.002  2.174  0.684  0.696  1.712 | 0.229  0.948  0.967  0.149  0.414  0.410  0.199 |
| N1 | Group  ISI  Side  Group x ISI  Group x Side  Condition x Side  Group x ISI x Side | 1, 35  1, 35  1, 35  1, 35  1, 35  1, 35  1, 35 | 1.201  0.480  0.073  0.403  4.012  1.066  4.377 | 0.281  0.493  0.789  0.529  0.053  0.309  <0.05 |
| P2 | Group  ISI  Side  Group x ISI  Group x Side  Condition x Side  Group x ISI x Side | 1, 35  1, 35  1, 35  1, 35  1, 35  1, 35  1, 35 | 0.264  1.184  1.222  0.327  0.302  0.003  0.966 | 0.611  0.284  0.277  0.571  0.586  0.960  0.333 |
| P2/2 | Group  ISI  Side  Group x ISI  Group x Side  Condition x Side  Group x ISI x Side | 1, 35  1, 35  1, 35  1, 35  1, 35  1, 35  1, 35 | 0.228  1.315  0.149  0.138  0.605  2.152  0.760 | 0.636  0.259  0.702  0.713  0.442  0.151  0.389 |

Table A. P50 amplitude test-conditioning stimulus gating ratios analysis using mixed model 3-way ANOVA. Key: Group, dexamethasone treatment group; ISI, interstimulus interval condition (500 ms vs. 250 ms); Side, recording side. Significant differences are underlined.

### S.2.7 Auditory Steady-State Response Statistics

| ASSR Stimulus Measure | Stimulus Rate | Side | Parameter | df | F-value | p-value |
| --- | --- | --- | --- | --- | --- | --- |
| Evoked Trial Power | 20 Hz | Left | Slope | 1, 176 | 0.206 | 0.650 |
|  |  |  | Intercept | 1, 177 | 16.303 | <0.0001 |
|  |  | Right | Slope | 1, 171 | 0.0002 | 0.990 |
|  |  |  | Intercept | 1, 172 | 2.206 | 0.139 |
|  | 40 Hz | Left | Slope | 1, 176 | 0.036 | 0.850 |
|  |  |  | Intercept | 1, 177 | 1.605 | 0.207 |
|  |  | Right | Slope | 1, 176 | 0.076 | 0.783 |
|  |  |  | Intercept | 1, 177 | 0.220 | 0.640 |
| Phase Locking Factor | 20 Hz | Left | Slope | 1, 176 | 0.205 | 0.651 |
|  |  |  | Intercept | 1, 177 | 4.985 | <0.05 |
|  |  | Right | Slope | 1, 171 | 0.053 | 0.818 |
|  |  |  | Intercept | 1, 172 | 0.596 | 0.441 |
|  | 40 Hz | Left | Slope | 1, 176 | 0.024 | 0.877 |
|  |  |  | Intercept | 1, 177 | 0.950 | 0.331 |
|  |  | Right | Slope | 1, 171 | 0.202 | 0.654 |
|  |  |  | Intercept | 1, 172 | 0.0003 | 0.986 |

Table B. Results from linear regression on measurements from auditory steady-state responses (ASSR) comparing slopes and means. Regression was performed on values averaged over 100 ms intervals for the first 500 ms after stimulus train start. Significant differences underlined.

| ASSR | Stimulus Rate | Side | Effect | df | F-value | p-value |
| --- | --- | --- | --- | --- | --- | --- |
| Mean Trial Power | 20 Hz | Left | Group  Time  Interaction | 1, 34  4, 136  4, 136 | 0.004  12.110  0.796 | 0.952  <0.0001  0.530 |
|  |  | Right | Group  Time  Interaction | 1, 34  4, 136  4, 136 | 0.009  12.900  1.072 | 0.927  <0.0001  0.373 |
|  | 40 Hz | Left | Group  Time  Interaction | 1, 34  4, 136  4, 136 | 0.024  6.403  0.832 | 0.877  <0.0001  0.507 |
|  |  | Right | Group  Time  Interaction | 1, 34  4, 136  4, 136 | 0.009  5.533  1.399 | 0.925  <0.001  0.238 |

Table C. Results from repeated measure 2-way ANOVA on mean trial power (MTP) from auditory steady-state responses (ASSR). Analysis was performed on values averaged over 100 ms intervals for the first 500 ms after stimulus train start. Significant differences underlined.

### S.2.8 ECG Parameter Statistics

| ECG Parameter | df | t-statistic | p-value |
| --- | --- | --- | --- |
| Heart Rate (BPM) | 34 | 0.774 | 0.444 |
| PR Interval (s) | 34 | 3.470 | <0.01 |
| P duration (s) | 34 | 3.487 | <0.01 |
| QRS Interval (s) | 34 | 2.049 | <0.05 |
| QT Interval (s) | 33 | 1.141 | 0.262 |
| Low Frequency/ High Frequency  Power Ratio ^A^ | 24 | 1.248 | 0.224 |

Table D. Differences in ECG parameters between dexamethasone and control animals. Comparisons using unpaired t-tests. Significant differences underlined. Key: ^A^ t-test using Welch’s correction for unequal variances.

# Supplementary Data References

1. Yates N, Robertson D, Martin-Iverson M, Rodger J. Auditory Brainstem Responses of Ephrin-A2-/-, Ephrin-A5-/-and Ephrin-A2A5-/-Mice. Audiology and Neurotology. 2014;19(2):115-26.

2. Knight RT, Brailowsky S, Scabini D, Simpson GV. Surface auditory evoked potentials in the unrestrained rat: component definition. Electroencephalography and clinical neurophysiology. 1985;61(5):430-9. doi: <http://dx.doi.org/10.1016/0013-4694(85)91035-1>.

3. de Bruin NMWJ, Ellenbroek BA, van Schaijk WJ, Cools AR, Coenen AML, van Luijtelaar ELJM. Sensory gating of auditory evoked potentials in rats: effects of repetitive stimulation and the interstimulus interval. Biological Psychology. 2001;55(3):195-213. doi: 10.1016/s0301-0511(00)00084-3.

4. Vohs JL, Andrew Chambers R, Krishnan GP, O'Donnell BF, Berg S, Morzorati SL. GABAergic modulation of the 40 Hz auditory steady-state response in a rat model of schizophrenia. The International Journal of Neuropsychopharmacology. 2010;13(04):487-97. doi: doi:10.1017/S1461145709990307.

5. Roach BJ, Mathalon DH. Event-Related EEG Time-Frequency Analysis: An Overview of Measures and An Analysis of Early Gamma Band Phase Locking in Schizophrenia. Schizophrenia Bulletin. 2008;34(5):907-26. doi: 10.1093/schbul/sbn093.

6. Sabeti M, Katebi S, Boostani R. Entropy and complexity measures for EEG signal classification of schizophrenic and control participants. Artificial Intelligence in Medicine. 2009;47(3):263-74. doi: <http://dx.doi.org/10.1016/j.artmed.2009.03.003>.
