# Supplementary material for: Effects of Neonatal Dexamethasone Exposure on Adult Neuropsychiatric Traits in Rats
Source: PLoS One. 2016 Dec 9;11(12):e0167220. doi: 10.1371/journal.pone.0167220 (PMC5147874; doi:10.1371/journal.pone.0167220)
Supplement: S3 File — Contains full statistical analysis and methods. (DOCX) [file pone.0167220.s003.docx]

#

## SUPPLEMENTARY DATA

## S.3 Real-time PCR

### S.3.1 Detailed Protocol

Auditory cortex samples were thawed on ice and total RNA extracted using 0.5 ml QIAzol Lysis Reagent per sample following manufacturer’s instructions (Qiagen). The RNA pellet was resuspended in 20 µl of RNase-free water and RNA concentration was determined using a NanoDrop*.* Total RNA (2 µg) was DNAse treated (RQ1 RNase-Free DNase; Promega) followed by denaturation at 65 °C for 10 min. Samples were reverse transcribed using 0.5 µl of Random Primers (500 ng/l) and MMLV reverse transcriptase (Promega). cDNA was purified using spin column clean up kits (Mo Bio) prior to amplification. Quantitative PCR was performed on a Rotorgene 6000 using standard curve method. All samples were processed in triplicate. Cycle parameters were: hold time 5 min at 95°C, with 45 cycles 10 s at 95°C and 30 s at 60°C. Melt curves were obtained from 61 to 95°C to confirm specificity of amplification. Primers were purchased from Quantitect primers (Qiagen) qPCR was performed using 3 reference genes (TBP (QT00198443), HPRT (QT00166768) and GAPDH (QT01658692), and 5 genes of interest (DRD1 (QT00386631), DRD2 (QT01081990), GAD1 (QT00194600) & GRIN2B (QT00184793), and TSC22 (QT01689632). Relative expression was calculated by using geometric mean of the reference genes.

### S.3.2 Quantitative Real-Time PCR Statistics

| Gene |  |  |  |
| --- | --- | --- | --- |
| t-test | df | t-statistic | p-value |
| DRD2 | 8 | 1.909 | 0.093 |
| GRIN2B | 8 | 1.752 | 0.118 |
| GAD1 | 8 | 0.854 | 0.418 |
| TSC22 | 8 | 2.633 | <0.05 |
| Mann-Whitney Test | Sum of Ranks | Mann-Whitney U | p-value |
| DRD1 | 26, 29 | 11 | 0.841 |

**Table A. Quantitative real-time PCR (qPCR) of gene expression results from the left auditory cortex.** Significant differences underlined.
